# Slow and continuous delivery of a low dose of nimodipine improves survival and electrocardiogram parameters in rescue therapy of mice with experimental cerebral malaria

**DOI:** 10.1186/1475-2875-12-138

**Published:** 2013-04-24

**Authors:** Yuri C Martins, Leah Clemmer, Pamela Orjuela-Sánchez, Graziela M Zanini, Peng Kai Ong, John A Frangos, Leonardo JM Carvalho

**Affiliations:** 1 Center for Malaria Research, La Jolla Bioengineering Institute, 3535 General Atomics Court Suite 210, 92121, San Diego, CA, USA; 2 Laboratory of Inflammation and Immunity, Professor Paulo de Góes Institute of Microbiology, Federal University of Rio de Janeiro, (UFRJ), Rio de Janeiro, Brazil; 3 Parasitology Service, Evandro Chagas Clinical Research Institute, Fiocruz, Rio de Janeiro, Brazil; 4 Laboratory of Malaria Research, Oswaldo Cruz Institute, Fiocruz, Rio de Janeiro, Brazil

**Keywords:** ECG, Adjunctive therapy, Experimental cerebral malaria, Nimodipine, Blood pressure

## Abstract

**Background:**

Human cerebral malaria (HCM) is a life-threatening complication caused by *Plasmodium falciparum* infection that continues to be a major global health problem despite optimal anti-malarial treatment. In the experimental model of cerebral malaria (ECM) by *Plasmodium berghei* ANKA, bolus administration of nimodipine at high doses together with artemether, increases survival of mice with ECM. However, the dose and administration route used is associated with cardiovascular side effects such as hypotension and bradycardia in humans and mice, which could preclude its potential use as adjunctive treatment in HCM.

**Methods:**

In the present study, alternative delivery systems for nimodipine during late-stage ECM in association with artesunate were searched to define optimal protocols to achieve maximum efficacy in increasing survival in rescue therapy while causing the least cardiac side effects. The baseline electrocardiogram (ECG) and arterial pressure characteristics of uninfected control animals and of mice with ECM and its response upon rescue treatment with artesunate associated or not with nimodipine is also analysed.

**Results:**

Nimodipine, given at 0.5 mg/kg/day via a slow and continuous delivery system by osmotic pumps, increases survival of mice with ECM when used as adjunctive treatment to artesunate. Mice with ECM showed hypotension and ECG changes, including bradycardia and increases in PR, QRS, QTc and ST interval duration. ECM mice also show increased QTc dispersion, heart rate variability (HRV), RMSSD, low frequency (LF) and high frequency (HF) bands of the power spectrum. Both sympathetic and parasympathetic inputs to the heart were increased, but there was a predominance of sympathetic tone as demonstrated by an increased LF/HF ratio. Nimodipine potentiated bradycardia when given by bolus injection, but not when via osmotic pumps. In addition, nimodipine shortened PR duration and improved HRV, RMSSD, LF and HF powers in mice with ECM. In addition, nimodipine did not increased hypotension or decreased the speed of arterial pressure recovery when used in rescue therapy with artesunate.

**Conclusions:**

These data show that slow and continuous delivery of lower doses of nimodipine improves survival of mice with ECM in rescue therapy with artesunate while showing a safer profile in terms of cardiovascular effects.

## Background

Human cerebral malaria (HCM) is a life-threatening complication during *Plasmodium falciparum* infections, contributing in large part to the estimated 900,000-1,600,000 malaria-related deaths worldwide [1]. Mortality usually exceeds 10% in controlled clinical trials, despite optimal treatment with intravenous anti-malarial drugs [2,3], and it is estimated that up to 25% of patients that survive an HCM episode suffer long-term neurological and cognitive deficits [4,5]. This scenario indicates that strategies targeting eradication of the parasite alone after HCM development have limitations and therefore adjunctive treatments are needed urgently [6-8].

*Plasmodium berghei* ANKA (PbA) infection of susceptible mouse strains is the best-studied experimental model of cerebral malaria (ECM) and is characterized by the development of neurological signs six to 12 days post-infection [7,9,10]. Mice with ECM show widespread cerebral vasoconstriction which markedly decreases blood flow in their brain [11]. High doses of the dihydropyridine calcium channel blocker nimodipine, given as an adjunctive therapy with artemether, prevents cerebral vasoconstriction and increases survival of mice with ECM when compared to control mice treated with the anti-malarial drug alone [11]. However, hypotension, bradycardia, arrhythmias, and eventually death may occur when nimodipine is given parenterally at high doses for humans [12,13] and these side effects could preclude the potential use of high doses of this drug to treat HCM, particularly in the most severe cases associated with shock and hypotension [14-16]. To solve this problem, the potential of alternative delivery systems and low doses for nimodipine in association with artesunate to rescue mice with late-stage ECM was analysed. The aim was to define optimal protocols and achieve maximum efficacy in increasing survival in rescue therapy while causing the least cardiac side effects. *Plasmodium berghei*-infected mice become hypotensive when presenting ECM signs [17], and therefore are a model particularly suited to study therapeutic approaches devised to address the most severe scenarios of this neurological complication.

Because little is known about the electrocardiographic (ECG) features of mice with ECM, the baseline ECG and arterial pressure characteristics of uninfected control animals and of mice with ECM and the response upon rescue treatment with artesunate associated or not with nimodipine were also determined. It is shown that continuous delivery of nimodipine by an implanted osmotic pump at low doses did not worse hypotension and improves ECG parameters and survival in rescue therapy of mice with ECM.

## Methods

### Mice, parasites, and infection

Eight- to ten-week-old female C57BL/6J mice were obtained from Jackson Laboratories (Bar Harbor, ME, USA). Mice were housed in groups of no more than five per cage and had free access to food and water. Mice were allowed three days to adapt to their new environment before experimentation. All experimental protocols were carried out in strict accordance with the recommendations in the Guide for the Care and Use of Laboratory Animals of the National Institutes of Health, reviewed and approved by the La Jolla Bioengineering Institute (LJBI) Institutional Animal Care and Use Committee. The *P*. *berghei* ANKA PbA-GFPcon 259cl2, which is a genetically modified parasite of clone cl15cy1 of the ANKA strain that expresses green fluorescent protein (GFP) constitutively during the whole life cycle, was used (a kind donation of MR4, Manassas, VA, USA; deposited by C J Janse and A P Waters; MR4 reagent number MRA-865). For each experiment a fresh blood sample was obtained from a passage mouse and a suspension containing 10^6^ parasitized red blood cells (pRBC) in 100μL was injected intraperitoneally (IP) in each mouse of the experimental groups. Parasitaemia, motor behaviour and rectal temperature were checked beginning on day 5 after infection. Parasitaemia was checked by using flow cytometry and quantified by counting the number of pRBCs in 10,000 RBC. After treatment, thin blood smears were made from a drop of tail blood and stained with Giemsa to distinguish dead from viable parasites as previously described [18]. The slides were examined under a light microscope at 1,000x magnification with an oil immersion lens (Nikon Eclipse E200). Parasitaemia was calculated by counting the number of pRBCs in at least 1,000 RBC. ECM was defined as the presentation of one or more of the following clinical signs of neurological involvement: ataxia, limb paralysis, poor righting reflex, seizures, rollover, and coma. Six behavioural tests (transfer arousal, locomotor activity, tail elevation, wire manoeuvre, contact righting reflex, and righting in arena) adapted from the SHIRPA protocol as previously described [18] were used to provide a better estimate of the overall clinical status of the mice during infection. Body temperature was monitored by using an Accorn Series Thermocouple thermometer with a mouse rectal probe (Oakton Instruments, Vernon Hills, IL, USA).

### Experimental design - survival studies

Mice presenting clinical signs of ECM were treated with artesunate (Sigma, St Louis, MO, USA, 32 mg/kg/day) or artemether (Artesiane 20 mg/mL – Dafra Pharma, Belgium, 25 mg/kg) in combination with either vehicle or nimodipine (Sigma, 0.5, 2.5, 4 or 12 mg/kg/day) in rescue treatments as previously described [18]. Nimodipine was diluted in ethanol: PEG 400:saline at 1:1:8 or 2:2:6 proportions and a total volume of 100μL/mouse per dose was given IP as a bolus injection or subcutaneously by a slow-delivery Alzet osmotic pump. Artesunate was diluted in the same vehicle as nimodipine. Artemether was already prepared in coconut oil. The same treatment protocols were used in the experiments to measure ECG and arterial pressure (see below).

### Preparation and implantation of osmotic pump

An Alzet osmotic pump (DURECT, Cupertino, CA, USA – model 1003D) with a constant delivery rate of 1 μL/hour for three days was filled with the appropriate solution (artesunate 32 mg/kg/day and nimodipine 0.5 or 2.5 mg/kg/day or vehicle as control – 100μL final volume). The pumps were primed in vitro in 0.9% sterile saline at 37°C for approximately four hours to ensure immediate delivery of the contents after implantation. Implantation of the osmotic pump was performed under sterile conditions. Mice were anesthetized using isofluorane and the primed pump was implanted subcutaneously on the back, slightly posterior to the scapulae. The delivery portal was implanted first to avoid interaction between the compounds delivered and the healing wound. Absorption of the compound by local capillaries results in systemic administration.

### ECG recordings

ECG recording sessions were performed during daytime. As described before [19], non-anesthetized mice were placed in a ecgTUNNEL® system platform (EMKA Technologies, France) and six-lead surface ECGs were acquired for 5 min using the Biopac MP-150 data acquisition system (Biopac Systems Inc, Goleta, CA, USA) at a rate of 2 kHz. The four sensors of the ECG platform, one for each paw, were coated with electrocardiographic gel and animals were physically restrained on the platform with a translucent size fitting half-tunnel. Paws were cleaned with 70% ethanol after data acquisition. Recordings were made over a period of 60–240 minutes and were analysed using EzCG analysis software (Mouse Specifics Inc, Quincy, MA, USA). ECG morphology, cardiac rhythm, and heart autonomic tone were analysed calculating the following parameters: a) heart rate (HR, frequency of heart depolarization-repolarization cycles, measured over the period of 1 min); b) R and SR waves amplitude (mean amplitude of signal measured from isoelectric line or from the signal minimum S line to peak of QRS, respectively); c) PR, QRS, heart rate corrected QT (QTc), and ST intervals duration; d) QTc dispersion (difference between largest and smallest values of QT intervals from the set of signals); e) heart rate variability (HRV, beat-to-beat variability in the heart rate in the time domain); f) root mean square of successive differences in NN intervals (RMSSD); g) total, low frequency (LF), and high frequency (HF) bands of the spectral power; h) Ratio of LF range power to HF range power.

### Arterial pressure measurements

Systolic, diastolic and mean arterial blood pressures were recorded in conscious mice using the CODA non-invasive tail-cuff system (Kent Instruments, Torrington, CT, USA) [20]. Mice were allowed to acclimate to the restrainer for 4 minutes prior to initiating arterial pressure measurements. At least 6 readings were taken from each animal at each time point. All parameters were recorded just before treatment (time 0) and at five time points after beginning of rescue treatments (3, 6, 24, 48 and 72 hours). Mice presenting ECM also had arterial pressures measured prior to infection (5 days before treatment).

### Statistical analyses

Results were expressed as means and standard errors of the mean (SEM) unless otherwise stated. When ECG and arterial pressure parameters were monitored over time, data collected before treatment were used as a baseline for each mouse and values obtained after treatment were converted and plotted as percentage of baseline. Then the area under the curve for each mouse in each treatment group was calculated and differences in the mean areas were compared. Statistical analysis was performed using student’s *t* test or one-way analysis of variance (ANOVA) with Tukey’s or Bonferroni’s post-tests when comparing whether one parameter varied between two or among three or more different treatment groups, respectively. When multiple concentrations of nimodipine were tested, post-tests to check for linear trend following one-way ANOVA were also performed. Kaplan-Meyer curves were used to represent survival data and the log-rank test was used to compare differences in survival curves. A p-value of less than 0.05 was considered significant. All statistics were calculated using GraphPad Prism 4.01 (GraphPad Software, San Diego, CA, USA).

## Results

### Continuous delivery of low doses of nimodipine improves survival of mice with ECM upon rescue treatment with artesunate

**Figure 1:**
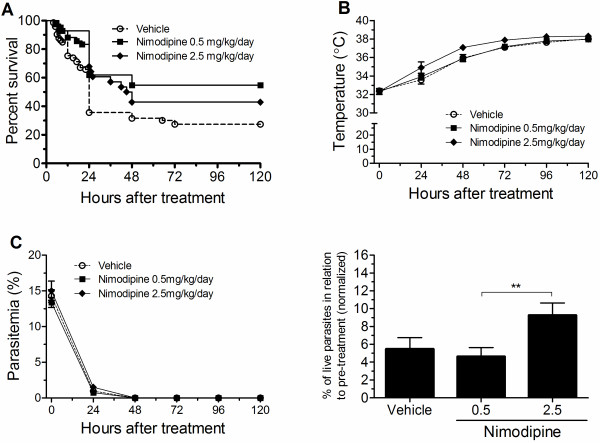
**Continuous delivery of low doses of nimodipine together with artesunate improves survival of ECM mice.** Cumulative survival (**A**), rectal temperature (**B**), and course of parasitaemia (**C**, left) of late-stage ECM mice treated with artesunate (32 mg/kg) together with slow continuous delivery via osmotic pumps of vehicle (n=73) and nimodipine at 0.5 mg/kg/day (n=42) or 2.5 mg/kg/day (n=28). Relative decay of viable circulating parasites 24 h after first dose of artesunate in relation to normalized parasitaemia before treatment was calculated to compare the efficacy of parasite clearance in each group (**C**, right). In panel **A**, p<0.05 when nimodipine-treated groups were compared with control mice treated with vehicle. **p<0.01. Results were pooled from three independent experiments.

It was previously shown that a bolus IP injection of nimodipine at 4 mg/kg or 12 mg/kg twice a day in association with artemether increased survival of mice with late-stage ECM [11]. However, whether continuous delivery of nimodipine at lower doses could improve survival of mice with late-stage ECM in association with an anti-malarial drug was never investigated. Mice presenting clinical signs of ECM were randomly assigned to treatment groups receiving either artesunate plus nimodipine at two different doses (0.5 or 2.5 mg/kg/day) or artesunate plus vehicle. Both groups of mice receiving artesunate plus nimodipine at 0.5 mg/kg/day and 2.5 mg/kg/day showed survival rates significantly higher than mice receiving artesunate plus vehicle (Figure 1A). The treatment with nimodipine at 0.5 mg/kg/day had a higher absolute risk reduction (ARR) and a lower number needed to treat (NNT) when compared with the 2.5 mg/kg/day dose (ARR = 27.36 vs. 15.46% and NNT = 3.65 vs. 6.46 for 0.5 and 2.5 mg/kg/day, respectively), indicating that the lower dose had a better performance to improve survival. There were no differences in motor behaviour scores, parasitaemia, and body temperatures at the beginning of treatment between groups receiving nimodipine or vehicle (Figure 1B-1C and Additional file 1). The overall rate of temperature recovery, indicated by the area under the curve of temperature, was greater in the group treated with nimodipine at 2.5 mg/kg/day and there was a trend to increase the pace of recovery with increasing of the dose (Figure 1B). The rate of parasite clearance in the nimodipine-treated groups did not differ from the vehicle-treated group. However there was a slower rate of parasite clearance in the group treated with 2.5 mg/kg/day of nimodipine when compared to the group treated with 0.5 mg/kg/day (Figure 1C). This difference in parasite killing may help to explain the better performance of the nimodipine at 0.5 mg/kg/day treatment. Preliminary trials with a lower dose of nimodipine (0.1 mg/kg/day) did not improve survival (data not shown).

### *Plasmodium berghei* ANKA infection induces ECG changes

**Figure 2:**
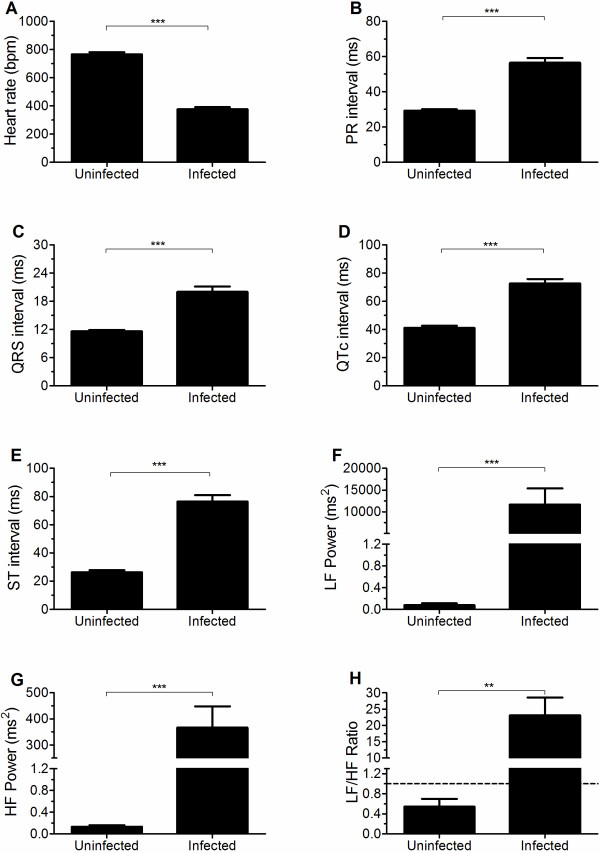
***Plasmodium berghei *ANKA infection induces ECG changes.** Heart rate (**A**), PR interval (**B**), QRS interval (**C**), QTc interval (**D**), ST interval (**E**), LF power spectral band (**F**), HF power spectral band (**G**), and LF/HF ratio (**H**) of uninfected (n=6) and PbA-infected mice presenting ECM signs (n=15). ECGs from ECM mice were measured between days 6 to 7 post-infection. **p<0.01, ***p<0.001.

**Figure 3:**
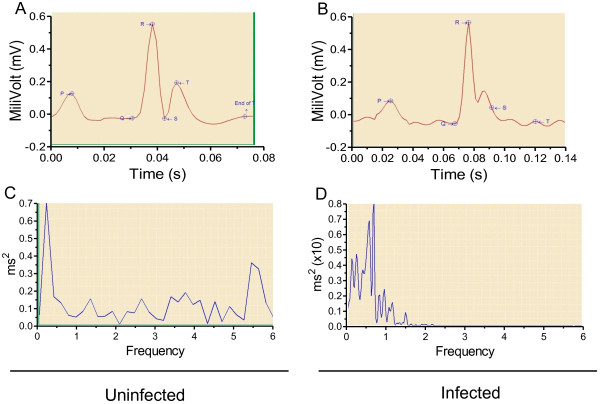
**Representative ECG changes induced by *Plasmodium berghei *ANKA infection.** ECG traces (**A**, **B**) and power spectra curves (**C**, **D**) from one uninfected (left) and one ECM mouse (right) on day 6 of infection. Note the global lengthening of ECG intervals in ECM mouse (**B**). In power spectral graphs, the total power of all the spectral components is represented by the area under the entire curve and is presented in units of square milliseconds/Hz. Area under peaks present between specific frequency bands provides information about low frequency (LF), and high frequency (HF) rhythms embedded within the HR pattern. LF lower bound is 0.03; LF upper bound is 1.5; HF lower bound is 1.51; and HF upper bound is 5.0. In **C**, uninfected mouse, total power is 0.64 ms^2^, LF power is 0.23 ms^2^, HF power is 0.26 ms^2^, and LF/HF ratio is 0.85 ms^2^. In **D**, ECM mouse, total power is 34,419.22 ms^2^, LF power is 33,774.93 ms^2^, HF power is 571.44 ms^2^, and LF/HF ratio is 59.1 ms^2^.

To determine whether PbA-infection per se could cause ECG changes in mice, ECGs from PbA-infected mice presenting ECM signs on days 6 to 7 post infection with the ones from uninfected mice were compared. Mice with ECM presented ~50% decrease in HR when compared to uninfected mice (Figure 2A). There were significant increases in PR, QRS, QTc, and ST intervals in ECM group, indicating a delay in current propagation through the heart conduction system and a lengthening in the depolarization, plateau, and repolarization phases of ventricles (Figures 2B–2E), which could at least partly explain the low HR observed in PbA-infected mice. Although all calculated intervals were increased in CM mice, the amount of increase was more intense in the ST interval. Mean R and SR wave amplitudes did not differ between CM and uninfected mice (Additional file 2A and 2B). Ten out of 15 mice with ECM presented sparse ventricular ectopic beats and two of them presented second degree AV block. Mice with ECM also showed significant hypothermia (Additional file 2C), which could partially explain the HR decrease and a global delay in current propagation through the heart. In addition, ECG parameters reflecting parasympathetic and sympathetic tonus to the heart were increased in mice with ECM as compared to uninfected controls. In fact, QTc dispersion, HRV, RMSSD, and total, LF, and HF powers were significantly increased in ECM mice (Figures 2F–2G and Additional file 2D-2G). Nonetheless, although parasympathetic and sympathetic inputs were increased in ECM mice, there was a predominance of sympathetic tone as shown by the increased LF/HF ratio (Figure 2H). Representative ECG traces and power spectra from uninfected and CM mice on day 6 of infection are shown in Figure 3.

### Bolus injection of nimodipine causes cardiac alterations in uninfected mice

**Figure 4:**
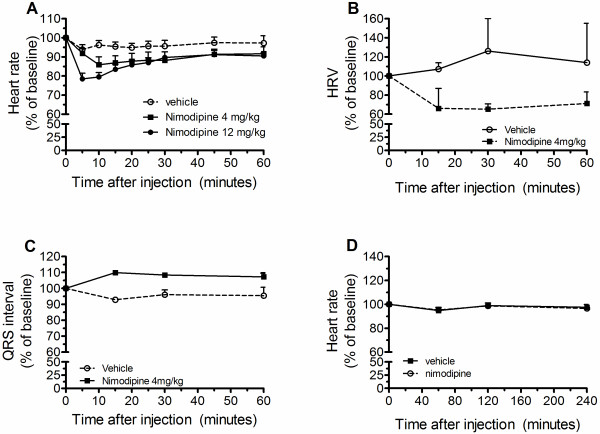
**Bolus injection of nimodipine causes ECG changes in uninfected mice.** Heart rate (**A**), heart rate variability (HRV, **B**), and QRS interval (**C**) variation over 60 min after IP injection of either nimodipine or vehicle in uninfected mice. (**D**) Heart rate variation over 240 min after slow and continuous delivery of either nimodipine or vehicle via an osmotic pump in uninfected mice. n ≥ 4 mice per group.

Nimodipine given IP at 4 and 12 mg/kg (bolus) in control, uninfected mice presented a trend for decreasing baseline heart rate compared to vehicle alone in a dose dependent manner over a period of 60 min (Figure 4A and Additional file 3A). The heart rate of nimodipine-injected mice remained lower than controls (vehicle-injected mice) for the length of the follow up (240 min). However, only when nimodipine was injected at 12 mg/kg was there a significant decrease in heart rate in relation to vehicle injection. Although nimodipine given IP at 4 mg/kg showed only a trend for decreasing heart rate of uninfected mice, there was a significant decrease in heart rate variability and an increase in QRS duration (Figures 4B and 4C and Additional files 3B and 3C), indicating that this lower dose also had cardiac effects. There was no significant difference in the other ECG parameters analysed between mice injected with nimodipine at 4 mg/kg and vehicle-injected mice. To determine whether the effects of nimodipine on heart rate could be abolished by decreasing the dose and changing the delivery rate, the effects of nimodipine given subcutaneously by osmotic pumps at 0.5 mg/kg/day were analysed. No changes in heart rate were observed with nimodipine delivered by this route in uninfected mice up to 240 min after implantation (Figure 4D and Additional file 3D).

### Slow subcutaneous infusion of nimodipine improves some ECG parameters and did not increase hypotension in mice with ECM

**Figure 5:**
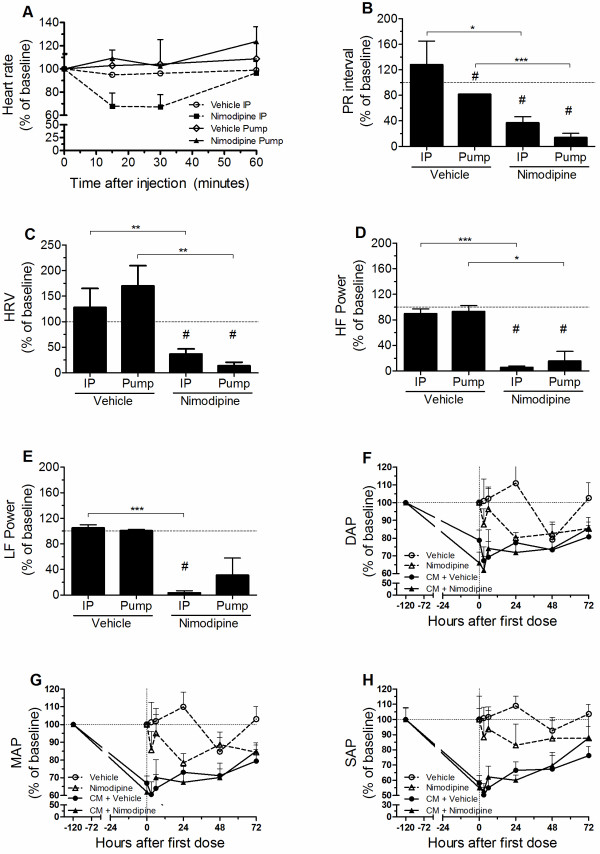
**Nimodipine improves ECG parameters and did not worse hypotension in ECM mice.** Heart rate variation (**A**) of ECM mice treated with either nimodipine or vehicle via IP injection or slow continuous delivery using osmotic pumps. PR interval (**B**), heart rate variability (HRV, **C**), HF power spectral band (**D**), and LF power spectral band (**E**) variation from baseline of ECM mice submitted to the same treatment schemes as in **A**. Except for **A**, values were measured before and 30 min after treatment in all groups. Diastolic (**F**), mean (**G**) and systolic (**H**) arterial pressures variation of uninfected and ECM mice treated with either nimodipine or vehicle given via osmotic pumps. Vertical dotted line indicates the moment of infection. Nimodipine was used at 4 mg/kg when used IP and at 0.5 mg/kg/day when used via an osmotic pump. n ≥4 mice per group. *p<0.05, **p<0.01, ***p<0.001, # indicates that mean is significantly different from baseline (horizontal dashed lines). DAP= diastolic arterial pressure; MAP= mean arterial pressure; SAP= systolic arterial pressure.

In mice with ECM, which were already bradycardic (see above) and hypotensive, nimodipine given IP at 4 mg/kg (bolus) caused a further ~30% decrease in HR between 15–30 min after injection, returning to values close to baseline after 60 min (Figure 5A and Additional file 4A). On the other hand, nimodipine given by osmotic pumps at 0.5 mg/kg/day caused no significant changes in HR when compared to vehicle-treated groups (Figure 5A and Additional file 4A).

As the effect of nimodipine given IP on HR peaked 15–30 min after injection, the effect of all treatments in other ECG parameters were compared at 30 min. Interestingly, both nimodipine treatment schemes (IP and osmotic pumps) significantly reversed the increases in PR duration, HRV, total power and HF power, an effect not observed in the vehicle-treated groups (Figures 5B-5D and Additional file 4B). Nimodipine given IP also significantly decreased the parameters reflecting parasympathetic input, LF power and RMSSD, but these effects did not reach significance when the drug was given via osmotic pumps (Figures 5E and Additional file 4C). There were no significant differences among the experimental groups in all other ECG parameters analysed (Additional files 4D-4J). Both nimodipine treatment schemes ameliorated the ECG parameter values, indicating that nimodipine can improve some of the cardiac changes induced by PbA infection. However none of ECG parameters modified by nimodipine treatment returned to levels similar to the ones obtained in uninfected mice.

One potential side-effect of nimodipine is hypotension. As ECM mice present hypotension, the adjunctive use of nimodipine during rescue treatment with artesunate could decrease even more the arterial blood pressure and delay the recovery of treated mice. To test this hypothesis, the effects of low doses of nimodipine given via osmotic pumps in the arterial pressure of uninfected and ECM mice were analysed. Infusion of low doses of nimodipine in uninfected mice decreased diastolic and mean arterial pressures to 80 – 90% of its baseline values, a significant decrease when compared to vehicle treated mice (Figures 5F-5G and Additional files 4K-4L). This effect was milder and not statistically significant in the systolic blood pressure (Figure 5H and Additional files 4M). ECM mice presented systolic, diastolic, and mean arterial pressures around 50 – 75% of their baseline values before starting treatment with artesunate (Figures 5F-5H). Infusion of nimodipine in ECM mice did not worse hypotension or delayed the improvement of systolic, diastolic and mean arterial pressures during rescue treatment with artesunate (Figures 5F – 5H and Additional files 4K – 4M).

## Discussion

It was previously shown that the dihydropyridine calcium channel blocker nimodipine, given via parenteral route (IP) as a bolus injection at high doses was able to improve the efficacy of artemether in rescuing mice with late-stage ECM from death [11]. Nimodipine is a drug administered by oral route to prevent cerebral vasospasm, a major complication of subarachnoid haemorrhages in humans [12]. However, nimodipine administration can cause potential deleterious cardiovascular side effects in humans, such as hypotension, bradycardia and arrhythmias when given intravenously even at therapeutic doses [12,21]. These possible cardiovascular side effects make the presence of hypotension and parenteral route of administration contra-indications for the use of nimodipine in the USA [22-24]. Hypotension may affect a significant fraction of all cerebral malaria cases and is strongly associated with poor outcomes in this population [14-16]; therefore, administration of nimodipine in this scenario might appear counterintuitive. Nevertheless, the present work showed that slow parenteral delivery of nimodipine at low doses caused no significant effects on cardiovascular parameters in normal mice and, more significantly, it actually ameliorated rather than worsened cardiac alterations in mice with late-stage ECM, reassuring its potential as adjunctive therapy. Indeed, mice with ECM present hypotension [17] and it is described in the present study that they also show bradycardia and ECG changes predisposing to arrhythmia, indicating that this host-parasite combination models the most severe and lethal forms of HCM. These findings provide better support for future clinical trials using nimodipine as adjunctive therapy in cerebral malaria.

Human CM encompasses a variety of clinical and pathological entities in which neurological involvement can be accompanied or not by several other complications such as respiratory distress, severe anaemia, acidosis, renal failure and shock [5,14,15,25]. Although significant bradycardia is not commonly present, ECG changes, cardiac arrhythmias and evidence of myocardial failure can also be observed in a fraction of patients with severe [26,27] and uncomplicated [28]*Plasmodium falciparum* malaria. ECG changes present in malaria patients have been shown to be mainly due to delayed conduction of various kinds and seem not to be related to death in affected patients [26-28]. This is the first study to report ECG changes present in mice with late-stage ECM. Previous studies monitoring ECG in PbA-infected mice without ECM showed bradycardia [29,30], but none of them described any changes in heart electrophysiology. The present work shows that mice with ECM present bradycardia, a global lengthening in the depolarization-repolarization cardiac cycle and an increase in the autonomic tone to the heart. ECG changes and bradycardia could be derived from both a directed effect of PbA infection in the heart and an indirect effect resulting from central nervous system dysfunction or metabolic disturbances. Direct damage to the cardiac conduction system and myocardium could explain the global enlargement of ECG intervals. In fact, the presence of cardiac lesions has been shown, ranging from myocyte cytoplasmic vacuolization to necrosis in mice infected with *Plasmodium chabaudi chabaudi*, *Plasmodium vinckei petteri* and *Plasmodium yoelii nigeriensis*[31]. Additionally, endomyocardial lesion and fibrosis have been described in PbA-infected mice [32], although direct PbA-induced heart pathology has not been confirmed in other studies [33].

Indirect cardiac effects due to central nervous system or metabolic dysfunction can also occur in mice with ECM and could account for the ECG changes observed. Mice with ECM develop hypothermia that is known to be associated with bradycardia and ECG changes reflecting slowing of myocardial conduction such as AV blocks, asystole, and increased PR, QRS and QTc intervals [34]. On the other hand, the increased autonomic tonus to the heart can reflect an autonomic dysfunction induced by PbA-infection that could be part of the ECM syndrome. In addition, other factors that can be present in late-stage ECM mice such as hypovolemia, hypoglycaemia, electrolyte disturbances, and acidosis are known to cause ECG changes and could also be related to these findings [34-36]. Further studies to analyse if the ECG changes described are specific of PbA-infected mice or also occur in other parasite-mouse combinations are a natural sequence of the present work. These studies could indicate if the central nervous system dysfunction present in mice with ECM cause or not the ECG changes described.

Nimodipine caused bradycardia, decreases HRV, and increase QRS duration in uninfected mice when given IP as a bolus injection at 4 mg/kg. On the other hand, ECG changes were minimized when nimodipine was given at 0.5 mg/kg/day via osmotic pumps. The cardiac side effects found in uninfected mice treated with nimodipine given IP can be explained by the decrease in the cardiac force of contraction and action potential conduction velocity associated with an increase in the myocyte effective refractory period caused by the blockage of voltage-dependent L-type Ca^+2^-channels [12]. Both the decrease in the treatment dose (from 4 mg/kg to 0.5 mg/kg/day) and the use of a slow and continuous delivery system contributed to prevent the cardiac side effects of nimodipine. In fact, it has been shown that, at the same dose, slow intravenous delivery of nimodipine does not differ from oral administration in efficacy and incidence of side effects when used to prevent vasospasm after subarachnoid haemorrhages [37].

Interestingly, when given IP, nimodipine increased bradycardia but also improved some ECG changes present in mice with ECM. This improvement can be related to its vasodilatory activity in brain vessels [11] rescuing normal brain physiology. This hypothesis could explain the shortening in the PR interval in treated mice, as nimodipine action in the heart actually tends to increase its duration when used in humans and healthy animals [12,13]. A role for artesunate in the changes observed is highly improbable as vehicle treated mice did not present any improvement in the ECG parameters analysed.

The present work also shows that the infusion of low doses of nimodipine decreased diastolic and mean arterial pressure in uninfected mice, but this effect was not observed in ECM mice. These findings show that low doses of nimodipine are safe to be given in hypotensive ECM mice and that the drug did not delay the arterial pressure recovery upon rescue treatment with artesunate.

## Conclusions

In the present study, the ECG alterations occurring in mice with late-stage ECM and the beneficial effect of nimodipine in some of these changes when the drug was used as an adjunctive therapy together with artesunate were characterized. It is also defined a safer dose and delivery system for nimodipine that increases survival of mice with ECM while having minimal cardiovascular side effects when compared with the treatment schemes given IP. These data indicate that nimodipine can be considered for further evaluation as a candidate for adjunctive therapy in CM. In addition, they also demonstrate that the PbA model can be used to analyse how drugs with cardiovascular effects can interfere with ECG changes induced by malaria.

## Abbreviations

ARR: Absolute risk reduction; DAP: Diastolic arterial pressure; ECG: Electrocardiography; ECM: Experimental cerebral malaria; HCM: Human cerebral malaria; HF: High frequency; HR: Heart rate; HRV: Heart rate variability; IP: Intraperitoneal; LF: Low frequency; MAP: Mean arterial pressure; NNT: Number needed to treat; PbA: *Plasmodium berghei* ANKA; pRBC: Parasitized red blood cells; QTc: Heart rate corrected QT interval; RMSSD: Root mean square of successive differences in NN intervals; SAP: Systolic arterial pressure

## Competing interests

The authors declare that they have no competing interests.

## Authors’ contributions

YCM carried out the statistical analyses, participated in the ECG data acquisition, and drafted the manuscript. LC carried out most of the ECG data acquisition and the experiments evaluating the efficacy of different nimodipine treatment schemes in rescuing mice with ECM. GMZ participated in the design of the study and in the ECG data acquisition. POS and PKO carried out arterial pressure data acquisition and analysis. JAF participated in the design of the study and critically revised the manuscript. LJMC conceived of the study, participated in its design and coordination, and helped to draft the manuscript. All authors read and approved the final manuscript.

## Supplementary Material

Additional file 1 **Motor behavioural score (A) of late-stage ECM mice treated with artesunate (32 mg/kg) together with slow continuous delivery via osmotic pumps of vehicle (n=73) and nimodipine at 0.5 mg/kg/day (n=42) or 2.5 mg/kg/day (n=28).** Areas under (AU) temperature curves in Figure 1B were calculated to compare the profiles of body temperature recovery in different treatment groups (B). There were no differences in the AU behaviour score curves among different groups. *p<0.05; arrow indicates the presence of a linear trend. Results were pooled from three independent experiments. Click here for file

Additional file 2 **ECG parameters not changed by *Plasmodium berghei *ANKA infection and temperature.** Mean R wave amplitude (A), mean SR wave amplitude (B), rectal temperature (C), QTc dispersion (D), heart rate variability (HRV, E), RMSSD (F), and total spectral power (G) of uninfected (n=6) and PbA-infected mice presenting ECM signs (n=15). ECGs from ECM mice were measured between days 6 to 7 post-infection. Rectal temperatures were measured just before ECG data was obtained. *p<0.05, **p<0.01, ***p<0.001. Click here for file

Additional file 3 **Areas under (AU) heart rate curves in Figure **2**A (A) were calculated to compare different treatment groups.** As HRV is already a measure of variation over time the mean variation over the 60-min period for each mouse was calculated and mean variation for each group was compared (B). _AU_QRS interval and _AU_heart rate curves in Figures 2C (C) and 2D (D), respectively. *p<0.05, **p<0.01, ***p<0.001; arrow indicates the presence of a linear trend. Click here for file

Additional file 4 **Variation in ECG parameters and Areas under (AU) heart rate and arterial pressure curves from uninfected and ECM mice treated with nimodipine or vehicle.** (A) _AU_Heart rate curves in Figure 5A. Total spectral power (B), RMSSD (C), QRS interval (D), QTc interval (E), ST interval (F), mean R wave amplitude (G), mean SR wave amplitude (H), QTc dispersion (I), and LF/HF ratio (J) variation from baseline of ECM mice treated with either nimodipine or vehicle via IP injection or slow continuous delivery using osmotic pumps. Values were measured before and 30 min after treatment in all groups. _AU_Diastolic (K), _AU_mean (L), and _AU_systolic(M) arterial pressure curves in Figures 5F – 5H, respectively. Nimodipine was used at 4 mg/kg when used IP and at 0.5 mg/kg/day when used via an osmotic pump. n ≥4 mice per group. *p<0.05, **p<0.01, ***p<0.001, # indicates that mean is significantly different from baseline (horizontal dashed lines). Click here for file
