# Supplementary figures and images for: Slow and continuous delivery of a low dose of nimodipine improves survival and electrocardiogram parameters in rescue therapy of mice with experimental cerebral malaria
Source: Malar J. 2013 Apr 24;12:138. doi: 10.1186/1475-2875-12-138 (PMC3642006; doi:10.1186/1475-2875-12-138)

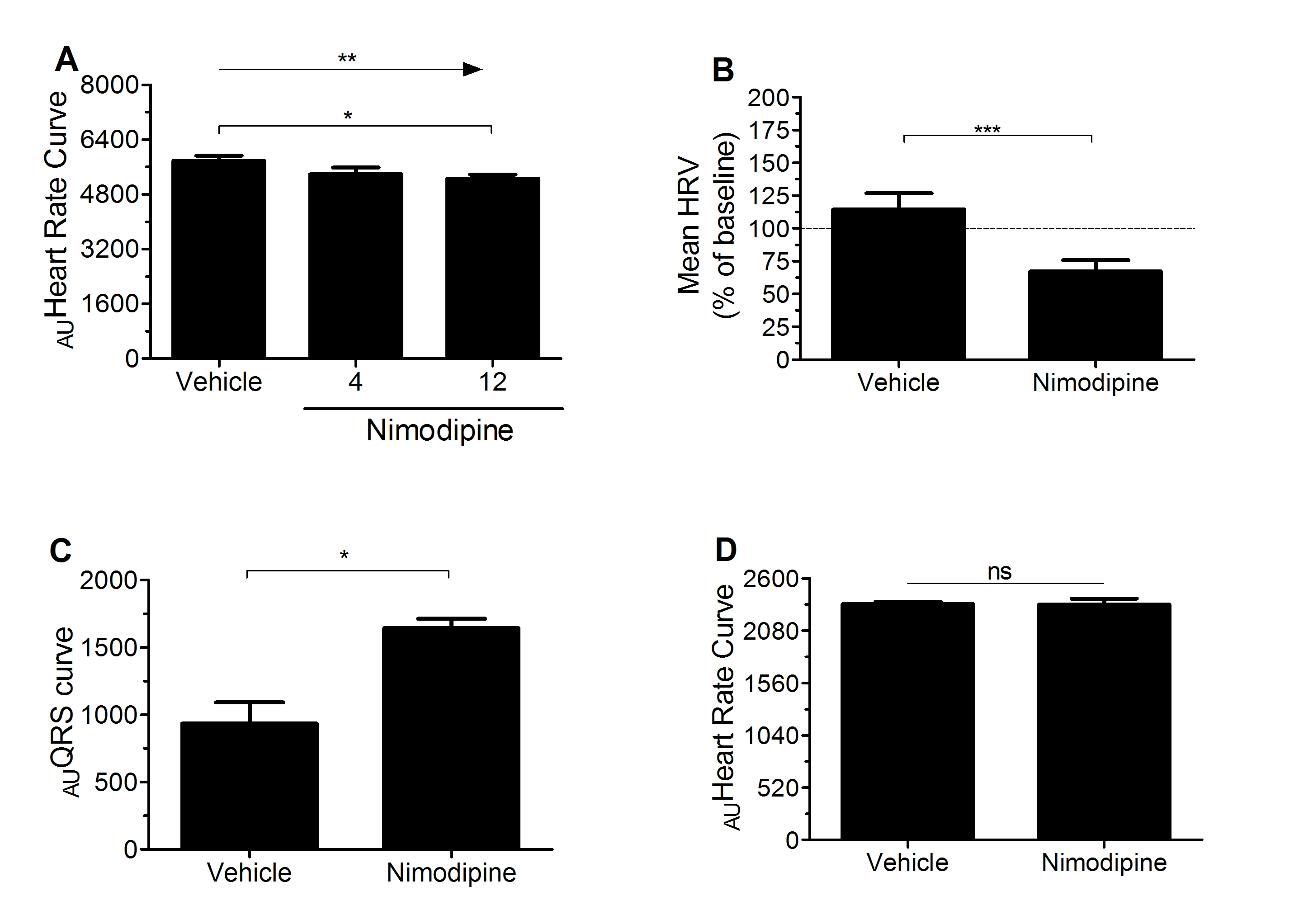

Supplement: Additional file 3 — Areas under (AU) heart rate curves in Figure 2A (A) were calculated to compare different treatment groups. As HRV is already a measure of variation over time the mean variation over the 60-min period for each mouse was calculated and mean variation for each group was compared (B). AUQRS interval and AUheart rate curves in Figures 2C (C) and 2D (D), respectively. *p<0.05, **p<0.01, ***p<0.001; arrow indicates the presence of a linear trend. [file 1475-2875-12-138-S3.tiff]
